# Fluorine-18-Fluorodeoxyglucose Positron Emission Tomography in Assessing Retroperitoneal Fibrosis: A Literature Review

**DOI:** 10.1155/2012/484052

**Published:** 2012-09-25

**Authors:** Giorgio Treglia, Maria Vittoria Mattoli, Francesco Bertagna

**Affiliations:** ^1^Institute of Nuclear Medicine, Catholic University of the Sacred Heart, Largo Gemelli, 00168 Rome, Italy; ^2^Department of Nuclear Medicine, University of Brescia, Brescia, Italy

## Abstract

*Background and Purpose*. Several studies have evaluated the role of fluorine-18-fluorodeoxyglucose positron emission tomography and positron emission tomography/computed tomography (FDG-PET and PET/CT) in diagnosing and assessing disease activity in patients with retroperitoneal fibrosis (RF). The aim of our paper is to perform a literature review on this topic. *Methods*. Scientific articles that evaluated the usefulness of FDG-PET and PET/CT in patients with RF were searched and discussed. *Results*. Eleven studies were found, and the main findings of these articles were described. *Conclusion*. FDG-PET and PET/CT are useful functional imaging methods for assessing patients with RF both in the diagnosis and in the treatment response evaluation. Moreover, further studies are needed to substantiate the role of FDG-PET and PET/CT in patients with RF.

## 1. Introduction

Retroperitoneal fibrosis (RF) is a chronic inflammatory disease, characterized by the presence of retroperitoneal inflammatory tissue, typically surrounding abdominal aorta and/or iliac arteries, and often leading to the involvement of adjacent structures, more frequently the ureters and inferior vena cava [1–7].

RF is a complex clinical entity still incompletely defined and with unclear etiology. Idiopathic RF (even reported as Ormond's disease) represents two thirds of all cases of RF. A true idiopathic form is present in any cases of RF in which no potential etiologic condition may be identified. The pathogenesis of the idiopathic RF appears today to be related to IgG4 autoimmune mechanisms “hyper-IgG4 disease”. Otherwise, RF in the presence of aortic atheromatous inflammation (atheromatous aortitis) has been included, more than twenty years ago, among the secondary forms, since this condition appears to be elicited by antigen-acting oxidized-LDL and/or ceroid, that are present within the atheromatous plaque. Etiology of other secondary RF refers to medications (drug-induced RF), infections, traumas, surgery, radiation therapy, and malignancies [1–7].

Clinical presentation of RF is usually characterized by constitutional symptoms and back or abdominal pain. Because of the presence of increased serum inflammatory markers levels and positive autoantibodies, and the frequent association with autoimmune diseases (such as Riedel's thyroiditis, sarcoidosis, inflammatory aneurysm, and autoimmune pancreatitis), some authors suspected that RF may result from autoimmune mechanisms [7] or, even, be considered as distinct autoimmune diseases [3, 8–11].

A frequent complication of RF is unilateral or bilateral ureteral obstruction, secondary to fibrosis and stenosis of the ureters [1, 4, 5]. Other described complications are small and large bowel obstruction and biliary obstruction [12].

The treatment of RF can be medical and/or surgical although a consensus has not been currently reached. The medical therapy is usually based on corticosteroids and immunosuppressive agents (such as tamoxifen) at different doses and various combinations on the base of disease activity and often leads to a substantial reduction of size of the retroperitoneal mass. The surgical procedure is required when severe obstructive complications are present [4, 5, 13–15].

However, determining the presence of active inflammation, and thus the potential for response to medical therapy, is crucial for the management of patients with RF and can be quite difficult, especially on the basis of only clinical parameters.

Most commonly used imaging methods in this pathologic entity are computed tomography (CT) and magnetic resonance imaging (MRI), despite their difficulty in discriminating between active and fibrotic lesions and in differentiating between RF and other retroperitoneal diseases such as tumors [16, 17].

Fluorine-18-fluorodeoxyglucose positron emission tomography and positron emission tomography/computed tomography (FDG-PET and PET/CT) are noninvasive functional imaging techniques which have become established tools in oncology [18]. Recently, FDG-PET and PET/CT have been proposed as potentially useful tools for study inflammatory diseases, such as large-vessel vasculitis (LVV) and atherosclerosis [19–23]. In fact, FDG is a glucose analogue that identifies areas of high-glucose metabolism. Since inflammatory cells, such as neutrophils, lymphocytes, and macrophages, have an increased glucose metabolism, high FDG uptake is revealed in vessel walls and retroperitoneal masses if inflammation is present [19–23].

Several studies have evaluated the role of FDG-PET and PET/CT in diagnosing, assessing disease activity, and monitoring RF. The aim of our paper is to review relevant published articles on this topic.

## 2. Literature Data about FDG-PET and PET/CT in Patients with Retroperitoneal Fibrosis

A comprehensive computer literature search of PubMed/MEDLINE was conducted in order to find relevant published articles on the role of FDG-PET and PET/CT in patients with RF. We used a search algorithm based on a combination of the following terms: (a) “retroperitoneal fibrosis” or “Ormond” and (b) “positron emission tomography” or “PET.” No beginning date limit was used; the search was updated until July 31st 2012. Studies investigating the role of FDG-PET and PET/CT in patients with RF were eligible for inclusion. Review articles or editorials, articles not in the field of interest of this paper, case reports, and preclinical studies were excluded.

Eleven relevant articles about the role of FDG-PET and PET/CT in patients with RF were found and discussed (Table 1).

In 2005, Salvarani et al. [24] evaluated the presence and extent of large-vessel inflammation in 7 patients with RF or other chronic periaortitis using FDG-PET. These authors found FDG-PET findings suggestive for LVV in patients with RF, demonstrating increased vascular radiopharmaceutical uptake involving abdominal aorta and/or common iliac arteries, which in some cases was also extended to the thoracic aorta and/or its branches [24].

In the same year, Vaglio et al. [25] used FDG-PET to evaluate the metabolic activity of residual masses at CT scan after immunosuppressive treatment in patients with RF. Six out of seven patients evaluated had no abnormal FDG uptake in the residual mass, and the seventh showed only moderate vascular FDG uptake. During the followup, none of the patients showed clinical, serological, or radiological signs of disease relapse. These findings suggested that FDG-PET is a sensitive mean to evaluate metabolic activity of residual masses in RF patients in clinical remission with detectable masses at CT scans [25].

In 2007, Nakajo et al. [26] reviewed six male patients with RF and confirmed that FDG-PET and PET/CT are useful tools for evaluating disease activity and extent of RF although these methods failed to differentiate between malignancy and RF. In fact, the authors performed early and delayed dual-time point imaging to examine the change of FDG uptake in RF, finding an increase of maximum standardized uptake value (SUVmax) at delayed images, which was similar to the pattern observed in malignancies. Abnormal FDG uptake was also noted in the mediastinum and in the pancreas of some patients, and the diagnosis of mediastinal fibrosis and autoimmune pancreatitis were made, respectively [26].

In 2008, Young et al. [27] reported FDG-PET-positive findings in three patients presenting with active RF. In one patient, repeat FDG-PET was performed following immunosuppressive therapy, with complete resolution of the retroperitoneal FDG uptake. The authors confirmed that FDG-PET may be a useful adjunct to anatomic imaging and serum inflammatory markers in distinguishing between active and inactive disease and in assessing the severity of inflammation in RF. This method may also be used in followup to assess therapeutic response if CT findings are unclear [27].

In 2010, Jansen et al. [28] evaluated whether FDG-PET was useful in the diagnostic and therapeutic evaluation of patients with RF treated with tamoxifen. At baseline, FDG-PET was positive in 20/26 patients. Patients with a positive FDG-PET scan had a higher serum inflammatory markers level (*P* = 0.02) and a larger mass size at CT scan (*P* = 0.01) compared with patients with a negative FDG-PET scan. FDG uptake decreased following treatment, in agreement with inflammatory markers reduction (*P* < 0.001), but not with CT-documented mass regression. These findings confirmed that FDG-PET may be useful to evaluate the severity and the extent of RF. Furthermore, these authors demonstrated that FDG-PET may be a valuable tool in assessing disease activity during or after treatment in patients with normal inflammatory marker levels and stable residual mass on repeated CT scans. Nevertheless, short-term FDG-PET followup did not accurately predict success of tamoxifen treatment [28].

In the same year, Piccoli et al. [29] supported the use of FDG-PET/CT not only for the diagnostic workup but also in the followup of patients with RF in order to tailor medical and surgical approaches. In particular, evaluating seven RF patients with FDG-PET/CT during the diagnostic workup and during followup, the authors found that this method may be useful to identify the best time for a safe removal of the renal stents (when no active disease is revealed by FDG-PET). FDG-PET/CT has been also useful to schedule for tapering of immunosuppressive therapy [29].

Recently, Pipitone et al. [30] presented four cases of RF with different etiology (one idiopathic RF and three secondary RF) and evaluated the role of FDG-PET in the workup of these different conditions. The authors highlighted that FDG-PET should be part of the investigations used to determine disease extent, activity, and response to therapy both in patients with idiopathic and secondary RF [30].

Ha et al. [31] recently investigated the clinical characteristics, laboratory findings, radiologic findings, treatment, and outcome in 27 Korean patients with RF. Only 6/27 patients have performed FDG-PET scan, and five out of six patients showed FDG-PET-positive findings. The authors suggested that FDG-PET may be a useful tool to detect active RF [31].

Bertagna et al. [32] assessed the feasibility and usefulness of FDG-PET/CT in 25 RF patients studied in two Italian nuclear medicine centres; 18/25 patients underwent PET/CT as initial evaluation study, 3/25 patients during followup, 3/25 during steroid therapy in absence of initial evaluation, and 1/25 to reevaluate the disease suspecting a recurrence. FDG-PET/CT was positive in 18/25 patients and negative in 7/25. Among the 10 patients who underwent a second study after steroid therapy, 6/10 showed complete metabolic response, 3/10 partial response, and one patient showed no significant FDG uptake reduction. The authors concluded that FDG-PET/CT is a suitable and useful tool in staging patients which are suspected to be affected by RF; moreover it could be very useful in treatment response evaluation [32].

Guignard et al. [33] evaluated the added value of contrast-enhanced CT (CE-CT) combined with FDG-PET as a one-stop diagnostic procedure for the assessment and followup of RF in 10 patients. These authors found that, unlike biologic and CT parameters, FDG uptake, was the most relevant parameter to measure severity of inflammation in RF. CE-CT allowed a better delineation of periaortitis and its extension to adjacent structures. The authors concluded that accurate determination of inflammation level in RF is significantly improved when FDG-PET/CT and CE-CT are performed in the same study and used for better delineation of areas of residual inflammation [33].

Lastly, Moroni et al. [34] in their recent prospective study on 22 patients with RF reported that FDG-PET/CT accurately discriminated active from inactive disease in about 94% of cases. A significant correlation (*P* < 0.01) was found among FDG uptake, and serum levels of inflammatory markers and among FDG uptake and analysis of contrast enhancement at CT images in the retroperitoneal mass. The authors concluded that FDG PET/CT may be considered an alternative imaging method for the assessment of different stages of RF [34].

## 3. General Remarks about FDG-PET and PET/CT in Patients with Retroperitoneal Fibrosis

The diagnosis of RF is based on clinical and radiological findings, and there are no established PET criteria for the diagnosis of this pathological entity. Many authors used a visual analysis of PET images for the evaluation of metabolic activity of RF. The visual analysis was usually performed using a four-point graded scale, based on the vessel-to-liver FDG uptake ratio (0: no uptake, 1: uptake less than that of the liver, 2: uptake equal to that of the liver, and 3: uptake greater than that of the liver); a visual score above 1 was usually considered a positive criteria for active RF.

However, standardization of FDG-PET studies by using semiquantitative analysis in order to achieve interchangeability in multicenter trials was recently recommended [35]. Six out of 11 articles included in this review have used a semiquantitative analysis based on the calculation of the SUVmax [26, 27, 29, 32–34]. The semiquantitative assessment of FDG uptake in the RF lesions is also useful for treatment response evaluation.

The added value of the hybrid technique PET/CT versus PET alone for RF diagnosis needs to be confirmed by further studies. In fact, only four articles evaluated the usefulness of FDG-PET/CT in patients with RF [29, 32–34]. Hybrid PET/CT allows the evaluation of disease activity and retroperitoneal mass size, as well as the localization of the inflammatory process in the same session. The main limitation of CT scan is the lack of discrimination between active and residual fibrotic tissue; nevertheless, CT may be useful in differentiating RF from atherosclerosis or in detecting morphological vascular alterations such as stenoses or aneurysms. Furthermore, a recent study suggested that the combination of FDG-PET and CE-CT performed in a single procedure would simplify the patient management [33]. This strategy could be more cost-effective and more accurate than separate studies acquired at different points in time although randomized prospective studies are required to validate this hypothesis [33].

Overall, FDG-PET and PET/CT seem to be useful methods both in evaluating disease extent and severity at the time of diagnosis and in assessing disease activity during or after treatment, mainly in patients with normal inflammatory marker levels and stable residual mass on repeated CT scans. Further prospective and multicentric studies with a larger patient population and cost-effectiveness studies are needed to correctly define the role of FDG-PET and PET/CT in patients with RF.

## 4. Conclusions

From this overview on the role of FDG-PET and PET/CT in patients with RF, we are led to conclude that these functional imaging techniques seem to be useful both in the diagnosis and in evaluating the treatment response. Further studies are needed to substantiate the role of FDG-PET and PET/CT in this setting.

## Figures and Tables

**Table 1:** Characteristics of the included articles.

Authors	Year	Country	Type of study	Patients evaluated by FDG-PET	Device used	Analysis of PET images	PET timing
Salvarani et al. [24]	2005	Italy	Retrospective	5 IRF, 2 PRF	PET	Visual	Before treatment
Vaglio et al. [25]	2005	Italy	Retrospective	7 IRF	PET	Visual	After treatment
Nakajo et al. [26]	2007	Japan	Retrospective	6 IRF	PET and PET/CT	Visual and semi-quantitative	Before treatment in 5 patients and after treatment in 1 patient
Young et al. [27]	2008	USA	Retrospective	3 IRF	PET	Visual and semi quantitative	Before treatment in 3 patients and after treatment in 1 patient
Jansen et al. [28]	2010	The Netherlands	Prospective	26 IRF	PET	Visual	Before treatment in 26 patients and after treatment in 18 patients
Piccoli et al. [29]	2010	Italy	Prospective	7 IRF	PET/CT	Visual and semi quantitative	Before and after treatment
Pipitone et al. [30]	2011	Italy	Retrospective	1 IRF; 3 SRF	PET	Visual	Before and after treatment
Ha et al. [31]	2011	Korea	Retrospective	6 IRF	PET	Visual	Before treatment
Bertagna et al. [32]	2012	Italy	Retrospective	25 IRF	PET/CT	Visual and semi quantitative	Before treatment in 18 patients and after treatment in 17 patients
Guignard et al. [33]	2012	Switzerland	Retrospective	7 IRF, 3 SRF	PET/CT	Visual and semi quantitative	Before and after treatment
Moroni et al. [34]	2012	Italy	Prospective	22 IRF	PET/CT	Visual and semi quantitative	Before and after treatment

Legend: IRF: idiopathic retroperitoneal fibrosis, SRF: secondary retroperitoneal fibrosis; PRF: perianeurysmal retroperitoneal fibrosis.
